# Editorial: Introducing Research Resource Identification Initiative at *eNeuro*

**DOI:** 10.1523/ENEURO.0046-16.2016

**Published:** 2016-01-04

**Authors:** Christophe Bernard

*eNeuro* is pleased to be part of the Research Resource Identification Initiative, a project aimed at clearly identifying the key resources used in the course of scientific research. This project helps address concerns about reproducibility by providing unique searchable identifiers, or Research Resource Identifiers (RRIDs), for critical reagents and tools. RRIDs link readers to external resources and enable search engines to return all papers utilizing a particular antibody, organism, or tool. Using RRIDs, authors can easily track all papers using various antibodies and assess how well the antibody works in different scenarios. *eNeuro* joins other neuroscience publications currently employing RRIDs. A complete listing of journals utilizing RRIDs can be found in Google Scholar or PubMed. This initiative is completely voluntary for *eNeuro* authors. RRIDs offer an important means for ensuring reproducible methods and providing critical data to help researchers identify suitable reagents and tools. We encourage all *eNeuro* authors to participate.

